# The Personalisation of Glioblastoma Treatment Using Whole Exome Sequencing: A Pilot Study

**DOI:** 10.3390/genes11020173

**Published:** 2020-02-06

**Authors:** Anne-Marie Garrett, Sarah Lastakchi, Christopher McConville

**Affiliations:** School of Pharmacy, Institute of Clinical Sciences, College of Medical and Dental Sciences, University of Birmingham, Edgbaston B15 2TT, UK; AXG773@student.bham.ac.uk (A.-M.G.); SXL117@student.bham.ac.uk (S.L.)

**Keywords:** glioblastoma, personalisation, whole exome sequencing, combination treatment

## Abstract

The molecular heterogeneity of glioblastoma has been linked to differences in survival and treatment response, while the development of personalised treatments may be a novel way of combatting this disease. Here we show for the first time that low passage number cells derived from primary tumours are greater than an 86% match genetically to the tumour tissue. We used these cells to identify eight genes that could be used for the personalisation of glioblastoma treatment and discovered a number of personalised drug combinations that were significantly more effective at killing glioblastoma cells and reducing recurrence than the individual drugs as well as the control and non-personalised combinations. This pilot study demonstrates for the first time that whole exome sequencing has the potential be used to improve the treatment of glioblastoma patients by personalising treatment. This novel approach could potentially offer a new avenue for treatment for this terrible disease.

## 1. Introduction

Glioblastoma multiforme (GBM) is the most common malignant tumour of the central nervous system (CNS) and is associated with poor prognosis, and with median survival rates of between 12 and 15 months [1,2]. GBM is characterized by its marked intratumoral heterogeneity [3,4,5,6] meaning that there are a number of genetically different clones, each of which is resistant to different treatments. The pre-existence of clones resistant to treatment has been demonstrated in various types of tumours [7,8]. These clones constitute the main cause of failure of targeted therapies and are responsible for tumour relapse after treatment. 

Personalised medicine has stemmed from an increased recognition of the importance of molecular heterogeneity within tumour types on drug-response and survival [9]. Multiple studies have been conducted to find molecular biomarkers for GBM that could be used as therapeutic targets as well prognostic or predictive factors [10]. For example, Epidermal Growth Factor Receptor (EGFR) amplification has been shown to lead to promotion of downstream signalling pathways that affect key processes in tumorigenesis such as proliferation and inhibition of apoptosis [11]. Reduced expression of the neurofibromin 1 (NF1) gene, as well as mutations in the Phosphatase and Tensin homolog (PTEN) gene and high expression of chitinase 3 like 1 (CHI3L1) and MET proto-oncogene, receptor tyrosine kinase (MET) have been shown to influence GBM growth [10]. 

A study into the effects of changes in growth control genes on patient survival supports the importance of isocitrate dehydrogenase (IDH) 1 mutations in glioblastoma [12]. In this study, mutated IDH1 was associated with better survival rates, as was reduced alpha thalassemia/mental retardation syndrome X-linked (ATRX) expression and/or lower expression of TP53 [12]. The possibility of PTEN as a biomarker for GBM is supported by Han et al., who conducted a meta-analysis of PTEN mutation and glioma survival rates, concluding that PTEN mutation is associated with poor prognosis [13]. 

Another key biomarker for GBM is the O6-methylguanine-DNA methyltransferase (MGMT) gene, which encodes a DNA repair enzyme that removes alkyl lesions from DNA [14,15]. Silencing of the promotor of this gene in GBM is associated with improved prognosis and increased response to alkylating chemotherapy agents [14]. A meta-analysis of the Telomerase Reverse Transcriptase (TERT) gene, a component of telomerase which is linked to immortalisation of cancer cells, highlighted it as being an important biomarker for gliomas [16]. Mutations in the promotor of this gene correlated with poor prognosis in glioma patients [16]. The association of the molecular profiles of GBM with prognosis highlights the need for developing effective personalised treatments, with the molecular make-up of the tumour informing the treatment regime for each individual patient. Such personalised treatments will require characterisation of the key genetic factors that influence a tumour’s response to different drugs. 

The current standard treatment regime for GBM patients involves surgery, radiotherapy and treatment with the chemotherapy drug temozolomide (TMZ) [11,17,18]. This regime remains largely ineffective, with recurrence common and survival rates poor. Consequently, other treatment options such as carboplatin, irinotecan and etoposide are being investigated [11]. Non-cytotoxic options are also being studied, such as targeted inhibitors, stem cell treatment, gene therapy and immunotherapy [11]. An example of a targeted inhibitor is Bevacizumab, a monoclonal antibody for Vascular Endothelial Growth Factor (VEGF)-A [17]. Other inhibitors are also being investigated, with targets including EGFR, PDGFR, phosphatidylinositide 3-kinases (PI3K) and Protein kinase B (PKB) [17]. 

With MGMT promotor methylation being identified as an important factor influencing response to TMZ it has consequently been used to stratify patients in clinical trials [14,15,18]. While this is an important step in associating molecular factors to drug response, more biomarkers for response to different therapies need to be identified. Oh et al. [19] performed a study to investigate if the personalised treatment of GBM based on the molecular subtype of the tumour was possible. While the researchers were able to demonstrate their proposed personalised treatments for each subtype were effective, the effectiveness of each treatment was not exclusive to each subtype. This suggests there are more molecular factors impacting drug response than those that classify the subtypes, and a fully non-targeted approach may provide more information. 

The aim of this study is to use Whole Exome Sequencing to analyse the genetic profile of six GBM tumour samples and associated cells then correlate this with each tumour sample’s response to treatment with the drugs captopril, celecoxib, copper glucomate, disulfiram, irinotecan, itraconazol, pitavastatin, temozolomide and ticlopidine. Each of these drugs has been shown either clinically or preclinically to have some efficacy against GBM. This data will then be used to develop personalised combinations from these drugs which will be assessed for their cytotoxicity and ability to reduce recurrence for each of the GBM samples.

## 2. Materials and Methods

### 2.1. Materials

Captopril, Celecoxib, Copper Glucomate, Disulfiram, Irinotecan, Itraconazol, Pitavastatin and Ticlopidine were purchased from LGM Pharma (Erlanger, KY, USA). Temozolomide was purchased from Sigma-Aldrich (Dorset, England). GBM tissue samples were retrieved from six patients who received resection surgery at the Queen Elizabeth Hospital, Birmingham, UK. 

### 2.2. Biopsy Collection, Dispersion and Culturing of the Patient Derived GBM Cells

The unfixed tumour core was collected directly from GBM patients undergoing craniotomies at the Queen Elizabeth Hospital in accordance with ethical approval (application number: 11-029) from the Human Biomaterials Resource Centre (HBRC). The samples were immediately placed in collection fluid and transported to the laboratory. The tumour tissue was subsequently immersed in HBSS (Invitrogen-Life technologies, CA, USA), sliced into 1 mm^3^ fragments and washed with HBSS to remove excess blood clots. Some of the tissue fragments were snap frozen in liquid nitrogen and stored at −80 °C for whole exome sequencing. The remaining fragments were then suspended in 30 mL of HBSS and digested with enzymes (Collagenase (0.25 mg/mL; Invitrogen-Life technologies, CA, USA), Pronase (0.5 mg/mL; Roche, Basel, Switzerland) and DNase (0.4 mg/mL; Sigma-Aldrich, Gillingham, UK)) for 30 min at both 37 °C and 4 °C with constant stirring. Any undigested material was then sieved using 100 μm pore nylon mesh and the suspension layered onto 2 × 12 mL Ficoll-paque density gradient cushions (Density: 1.077 ± 0.001 g/mL; GE healthcare life sciences, IL, USA) and centrifuged at 400 *g* for 30 min at room temperature. The tumour cells that settled as a band at the interphase were siphoned off, while the blood cells, which formed a pellet, were removed. An amount of 15 mL of HBSS was then added to the tumour cells and the solution centrifuged for 5 min at 1200× *g*. The supernatant was removed and the pellet re-suspended in 1 mL of HBSS ready for the viability check. Cell viability was determined using the Trypan blue exclusion method, with viability scores between 98% and 100%. The cells were snap frozen in liquid nitrogen and stored at −80 °C until needed.

### 2.3. Whole Exome Sequencing of the GBM Tumour Tissue and Cells

DNA was extracted from the tissue fragments and corresponding cells using a modified DNEasy protocol (Qiagen, Hilden, Germany). Briefly the tissue fragments and cells were incubated with ATL buffer and proteinase K overnight at 56 °C with intermittent vortexing. Samples were then processed according to the manufacturer’s instructions. Library preparation was performed using Illumina TruSeq exome library preparation kit according to the manufacturer’s instructions with the following modifications: 300 ng of material was used as starting input, no size selection was performed after end repair and DNA fragments were amplified with 12 cycles of PCR. Enrichment was performed using a bead ratio of 0.8, then samples were combined into pools of 3 plex for coding exome probe hybridisation and subsequent clean up. Then, 10 cycles of amplification were performed to enrich the final libraries which were then pooled into 1 final 12 plex library. Sequencing was performed on an Illumina NextSeq 550 75 cycles paired end reads high output flow cell.

### 2.4. Determination of the Cytotoxicity of Each Individual Drug and Drug Combination Against the Patient Derived GBM Cells

The cells were thawed and seeded at 2 × 10^5^ cells/cm^2^ in culture media compromised of 1:1 ratio of Dulbecco’s Modified Eagle’s Medium with L-glutamine and sodium bicarbonate (Sigma-Aldrich, Gillingham, UK) supplemented with 10% Fetal Bovine Serum, 100 μM sodium Pyruvate, 0.05 mM Non-essential Amino Acids and 1% Antibiotic-Antimycotic containing penicillin, streptomycin and fungizone (Invitrogen-Life technologies, CA, USA) and incubated at 37 °C, 5% CO_2_ with fresh media added every 2 days. Once confluent the cells were split by removing the culture media and adding trypsin (Sigma-Aldrich, Gillingham, UK) (2.5 mL for 75 cm^3^ flasks and 1.5 mL for 25 cm^3^ flasks) to the flask to detach the cells. An amount of 10 ml of fresh culture media was added to the flask and subsequently transferred to a centrifuge tube. The suspension was centrifuged for 3 min at 1000 rpm, the supernatant removed, and the pellet re-suspended in culture media and incubated at 37 °C, 5% CO_2_. The cells were split a further two times, so that we were working with passage number 3. The cells were split into two groups with one group sent for whole Exome sequencing and the others plated for cell viability studies.

The cells were plated onto 96-well flat-bottomed microtitre plates and cultured in the presence of 200 μL of cell culture media containing varying concentrations (3.9, 7.8, 15.6, 31.25, 62.5, 125, 250, 500, 1000, 10,000, 100,000 nM) of the individual drug for 5 days when cytotoxicity testing was performed using the standard 3-(4,5-Dimethylthiazol-2-yl)-2,5-Diphenyltetrazolium Bromide (MTT) assay. For the drug combinations, a solution containing each of the drugs was prepared and then added to the cell culture media and the cells were cultured for 5 days when cytotoxicity testing was performed. To investigate the influence of both individual drugs and drug combinations on recurrence, the cells were cultured for either 3, 5, 7, 9 or 11 days when cytotoxicity testing was performed. All experiments were performed in triplicate.

## 3. Results

### 3.1. Comparison of Variants Between GBM Tumour Tissue and Associated Cells

**Figure 1:**
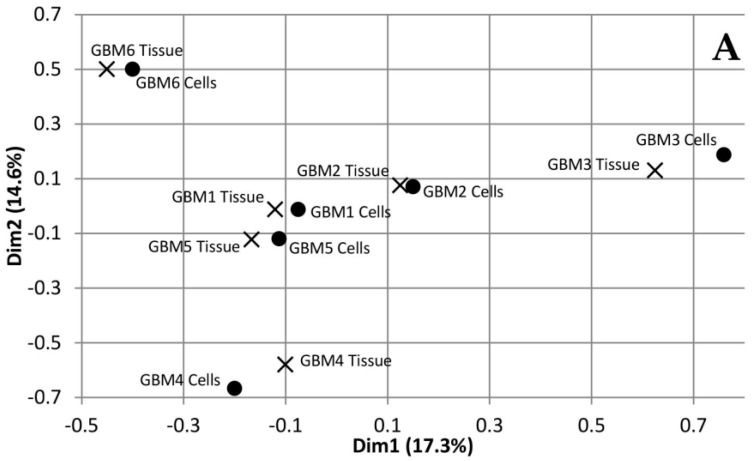

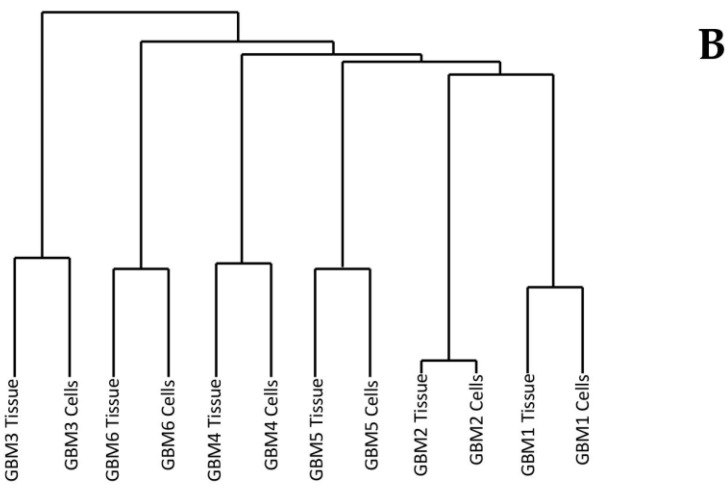
Dimensions 1 and 2 from Multiple Correspondence Analysis (MCA) of genomic variants in Glioblastoma multiforme (GBM) tumour tissue and cell samples (**A**). Dendrogram of GBM samples produced from hierarchical clustering of component values from dimensions 1-10 of the MCA (**B**).

Multiple Correspondence Analysis (MCA) and hierarchical clustering were performed using the FactoMineR package in R to assess the similarity between each of the tumour tissue samples as well as the similarity between the tumour tissue and the associated cells. The MCA plot showing dimensions 1 and 2 (Figure 1A) demonstrates that GBMs 1, 2 and 5 cluster together in the centre, while GBM 3, 4 and 6 are not only isolated from the other three tumours but also each other. This data demonstrates the inter-tumoural heterogeneity of GBM, as from the six samples analysed, only three are closely related molecularly while the other three are not only molecularly distinct from each other but also the others. Furthermore, the cells that were cultured from the tumour tissue cluster closely to their associated tissue, which demonstrates that they are molecularly similar to the tissue. The dendrogram produced from hierarchical clustering (Figure 1B) further demonstrates that the cell and tumour tissue samples from the same patient are molecularly similar to each other compared to samples from other patients. This data demonstrates that our cell culturing protocol did not significantly alter the molecular nature of the cells. Furthermore, comparing the fold change of the 24 most commonly mutated genes in GBM on the Catalogue of Somatic Mutations in Cancer (COSMIC) database in both the tumour tissue and associated cells demonstrated that our cells, at passage number 3, had a greater than 86% representation of the original tumour tissue and supports their use in personalisation of treatment as a representation of the tumour.

### 3.2. Selection of Mutated Genes for Personalisation

**Figure 2:**
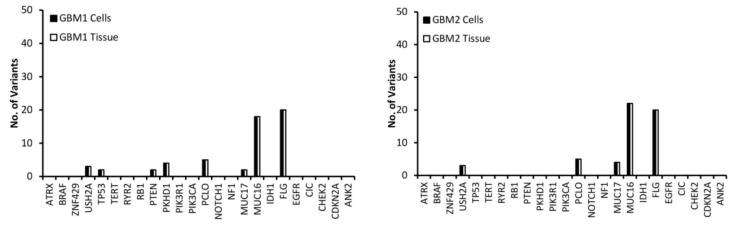

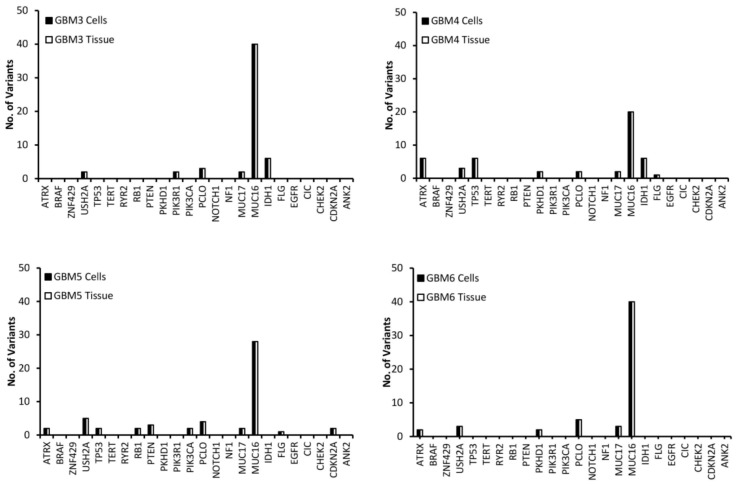
The number of exonic, nonsynonymous variants for the top 25 most frequently mutated genes in GBM according to the Catalogue of Sonic Mutations in Cancer (COSMIC) database in both the tissue and corresponding cells.

Variants in the top 24 most commonly mutated genes in GBM on the Catalogue of Somatic Mutations in Cancer (COSMIC) database were analysed for function and exonic function to compare the genetic profiles of the samples and to identify possible variant genes of interest. Our focus was on exonic, nonsynonymous variants as these are the only variants likely to have an impact on tumour function and growth. Figure 2 presents the number of variants in each of the 24 genes for both the tissue and cells for each of the GBM samples. The first observation is that all of the genes have the same number of variants in both the tissue and cell sample from the same patient, further demonstrating that the cells have a high molecular representation of the corresponding tissue supporting their use in the personalisation of GBM treatment. The second observation is that there is no correlation between the number of variants or the gene these variants occur in across all of the patients, further demonstrating the inter-tumoural heterogeneity of GBM and the need for personalisation of treatment.

A low (less than 10) number of variants were detected in all samples for the USH2A, PCLO and MUC17 genes (Figure 2). MUC16 has a significantly higher (between 20 and 40) number of variants compared to the other genes (Figure 2), which is not surprising as Tan et al. have demonstrated that it has a high mutation frequency due it being a large protein with a low expression level and late replication timing during the cell cycle [20]. There were 20 variants of FLG in GBM 1 and 2 (Figure 2). A low number of ATRX variants were seen exclusively in GBM 4, 5 and 6 (Figure 2), however, according to the pathology data (Table 1) only GBM 4 had a mutation of the ATRX gene. This makes sense as only GBM 4 had a mutated IDH gene and ATRX mutations in brain tumours are frequently detected with IDH mutations [21]. A low number of variants of TP53 were exclusive to GBM 1, 4 and 5 (Figure 2), with only GBM 4 having a loss of p53 function (Table 1) as a result. GBM 4 had 6 variants of TP53, while GBM 1 and 5 had 2 (Figure 2). Variants of PTEN were seen exclusively in GBM 1 and 5, while variants of PKHD1 were seen in GBM 1, 4 and 6 (Figure 2). Only GBM 3 and 4 had variants of the IDH gene (Figure 2), which correlates with the pathology data as both of these samples expressed the mutant protein IDH1 variant R132H (Table 1), which is associated with a better response to treatment (12). GBM 3 was the only sample to have variants of the PIK3R1 gene, while GBM 5 was the only sample to have variants of the RB1, PIK3CA and CDKN2A genes (Figure 2).

To make personalisation easier, it was decided to only focus on those genes that may be associated with treatment response. To identify the function of the genes, Gene Ontology (GO) terms were obtained from the gene2go file available on the NCBI FTP site [22,23,24]. From the GO processes the function of each gene is summarised in Table 2, with the most promising candidate genes associated with treatment response being those involved in transcriptional regulation (ATRX), tumour suppression (TP53, PTEN and CDKN2A), proliferation and survival (IDH1, PKHD1, PIK3R1 and PIK3CA). These genes will be used to personalise treatment.

Mutations in the ATRX, TP53, PTEN, IDH1, PIK3R1 PIK3CA genes are known to be cancer drivers in GBM according to the Cancer Genome Atlas Programme (TCGA). It is well understood that a wild type IDH gene makes GBM more aggressive [25,26,27,28], while a wild type ATRX gene makes GBM less sensitive to DNA damaging agents such as IRN and TMZ [29]. The p53 gene is mutated in 84% of GBM patients, with these mutations responsible for GBM cell invasion, migration, proliferation, evasion of apoptosis, and cancer cell stemness [30]. Therefore, variants in these genes make excellent candidates for the personalisation of GBM treatment. Variants in the PTEN gene could also be useful in the personalisation of GBM treatment as mentioned previously; Han et al. established a relationship between PTEN mutations and GBM patient survival [13]. Jiang et al. demonstrated that PTEN mutations are associated with therapeutic resistance [31]; while Benitez et al. demonstrated that PTEN regulates GBM oncogenesis [32]. Mutations in the PIK3R1gene have been shown to promote the growth and formation of gliomas [33], while PIK3CA missense mutations have been shown to promote GBM pathogenesis [34].

Mutations in the PKHD1 and CDKN2A genes are not known to be cancer drivers in GBM according to the TCGA. However, Draaisma et al. demonstrated that mutations in the PKHD1 gene is associated with poor prognosis in glioma patients [35], while studies have shown that mutations in the CDKN2A gene results in a loss of expression in the p16 protein, an inhibitor of cell cycle progression, and is associated with a significantly shorter survival in GBM patients [36,37,38].

USH2A, MUC16, MUC17 and FLG were not used in the personalisation of treatment as according to GO they have no influence on the growth or survival of GBM (Table 2). Furthermore, we could find no conclusive evidence in the literature that linked mutations in these genes to the growth and survival of GBM. Even though studies have shown that mutations in the PCLO gene could be involved in GBM growth and survival [39], it was removed from the personalisation process as every GBM sample had variants of this genes (Figure 2), making it difficult to link it to drug response.

### 3.3. Drug Response vs. Variant

Based on background research we selected two cytotoxic drugs (Irinotecan and Temozolomide) and seven non-cytotoxic drugs (Pitavastatin, Disulfiram, Copper Glucomate, Captopril, Celecoxib, Itraconazole and Ticlopidine) to use in the drug response studies. These drugs were selected because they are pharmacologically well characterized and had evidence for interfering with a recognized, well-characterized growth promoting element of GBM [40,41,42] and when combined had a reasonable likelihood of concerted activity against key biological features of GBM growth. An additional reason for investigating the non-cytotoxic drugs is because they have a low likelihood of adding to a patient’s side effect burden. Given the fact that in personalised GBM treatment we would be administering a combination of drugs, it will be important to use drugs that do not add to the side effect burden of the patient.

**Figure 3:**
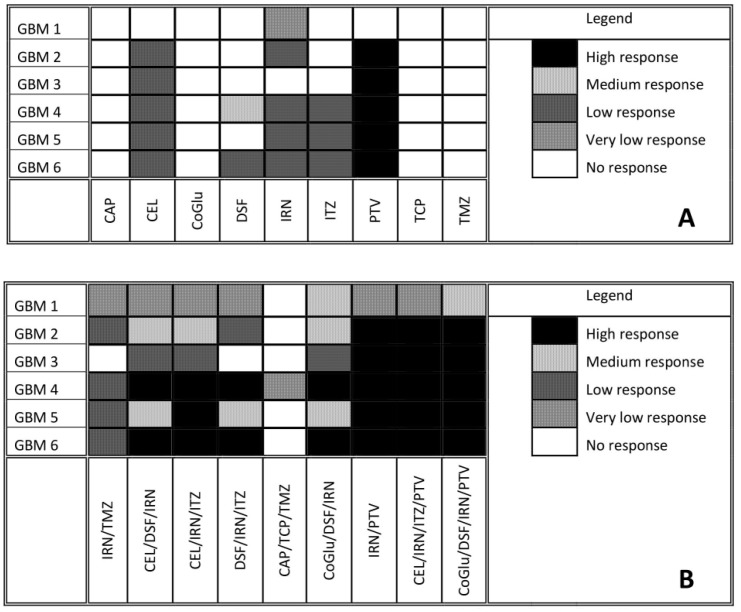
Response of GBM cells to each individual drug after five days of incubation (**A**). Response of GBM cells to a series of personalised, random and clinically relevant drug combinations (**B**). If the drug failed to achieve an IC_50_ then the response was defined a no response. A high response corresponds to an IC_50_ value between 2 and 3 Log nM, a medium response between 3 and 4 Log nM, a low response between 4 and 5 Log nM and very low above log 5 nM.

To find genes that may be associated with a patient’s response to the selected drugs, cell viability testing was performed. Variants were classed as being associated with drug response if they were present exclusively in all samples that responded to the treatment. Samples were considered unresponsive to treatment if they failed to reach an IC_50_ value. A log IC_50_ value between 2 and 3 nM was classed as a high response, a value between 3 and 4 nM as a medium response, a value between 4 and 5 nM as a low response and a value above 5 nM as a very low response. Figure 3A shows the response of each GBM sample to all nine drugs. Many of the drugs tested produced either a low or no response in all of the GBM samples. The two most interesting observations from Figure 3A are that: (1) TMZ produced no response in any of the GBM samples, even though only GBM 2 and 4 had an unmethylated MGMT, and (2) PTV produced a high response in all of the GBM samples except for GBM 1. Only IRN produced a very low response in GBM 1, which is not surprising given that it has a wild type IDH gene (Table 1) making it more aggressive [25,26,27,28]. This coupled with its wild type ATRX gene (Table 1), which makes it less sensitive to DNA damaging agents such as IRN and TMZ [29], would make it a very aggressive GBM. The lack of response, especially from TMZ, highlights the problem with the current treatment regime for GBM and the need for improved therapy options.

CAP, CoGlu and TCP did not produce a response in any of the samples, while CEL, a non-steroidal anti-inflammatory drug, which has been shown to stop the growth of GBM tumour cells by blocking the enzymes necessary for their growth, produced a low response in GBMs containing variants in the ATRX, TP53, PTEN, PIK3R1, PIK3CA, IDH 1 and CDKN2A genes (Figure 3A). GBMs containing variants of the ATRX, TP53, PTEN, PKHD1, PIK3CA and CDKN2A genes responded to treatment with IRN (Figure 3A), a semi-synthetic pro-drug [43], who’s active metabolite acts as an inhibitor of the Topoisomerase I group of enzymes [44]. ITZ, an antifungal that has been shown to suppress the growth of GBM through the induction of autophagy, produced a low response in GBMs with variants in the ATRX, TP53, PTEN, PIK3CA and CDKN2A genes (Figure 3A). Even though DSF produced a medium response in GBM 4 and low response in GBM 6 (Figure 3A) we could not identify any specific gene involved.

The fact that PTV produced a high response in five out of the six GBM samples (Figure 3A) makes it difficult to identify any specific genes that it may target, which in turn makes personalisation difficult. Jiang et al. demonstrated that PTV had an IC50 value of between 1.26 and 55.65 M across a range of GBM cells [40], while our data has shown that PTV has an IC50 value between 0.1 and 1mM for GBMs 2 to 6. Jiang et al. suggested that PTV worked against GBM cells by inducing autophagy via the LC3 pathway [40]. The Microtubule-associated protein 1A/1B-light chain 3 (LC3) is encoded by the gene MAP1LC3B gene; however, we detected no variants of this gene in any of our samples and thus we would have expected PTV to have a similar response across all samples. In another paper Jiang et al. demonstrated that PTV worked via targeting the mevalonate synthesis pathway, which is involved in the synthesis of cholesterol [45]. GBM survival depends on cholesterol and thus its depletion via blocking of the mevalonate synthesis pathway would lead to cell death [46]. Again, we detected no variant in any of the genes (ACAT, HMGCS1, HMGCR, PMVK, MVK, MVD and IDI) involved in the mevalonate synthesis pathway. Furthermore, if this pathway is involved in PTV’s mechanism of action we would have expected all samples to have similar responses. GBMs have been shown to have higher lipid levels compared to lower grade gliomas, while the more aggressive GBMs have been shown to have higher lipid levels than less aggressive GBMs [47]. According to the pathology data (Table 1) GBM 1 is very aggressive. Therefore, we hypothesise that the highest dose of PVT administered was insufficient to reduce the lipid levels of GBM 1 to levels were its survival would be compromised. Our observations support the hypothesis that PTV works by reducing the lipid levels in GBMs to levels where they can no longer survive and will only work on the least aggressive GBMs that already have lower lipid levels.

### 3.4. The Influence of Personalisation on Cell Viability

To aid with the personalisation of treatment the cell viability data from Figure 3A and the variants associated with drug response was summarised in Table 3. Before we began the personalisation process, and to act as a control combination, we tested TMZ and IRN, which is a combination that has been used in the clinic with promising results [48,49]. However, Figure 3B demonstrates that for this group of patient samples combining TMZ with IRN provided no additional benefit compared to IRN alone (Figure 3A). Therefore, in a clinical setting these patients would suffer the additional side-effects of TMZ with no treatment benefit. Using the data from Table 3 we selected three, three-drug combinations that would target as many of the candidate genes associated with treatment response as possible. In the initial stages of personalisation, we decided not to use PVT as it gave a high response to all of the GBM samples, except for GBM 1, and we felt that this would obscure any additional benefit seen from personalisation. Furthermore, to confirm our hypothesis that personalising treatment is more effective than non-personalisation of treatment and to demonstrate that cell death was not due to a ‘cocktail’ of ‘random’ drugs we used a combination of drugs (CAP/TCP/TMZ) that had no response when used individually.

When we combine CEL and DSF with IRN we see an increased response across all GBM samples except for GBM 1 and 3 (Figure 3B). This is not surprising as based on Table 3 this combination targets all eight of the candidate genes associated with GBM growth. For example, CEL, DSF and IRN all target candidate genes that are present in GBM 4 and 6 and we see a high response, were as only CEL and IRN target the candidate genes that are present in GBM 2 and 5 and in this case we see a medium response. For GBM 1 only IRN targets the candidate genes that are present, and thus we see no increase in response when compared to IRN alone (Figure 3A). The same observation is made with GBM 3 (Figure 3) were only CEL targets the mutated genes present (Table 3).

When we replace DSF with ITZ in the combination we also see and increased response across all GBM samples, however, the pattern of increase is different (Figure 3B). For example, with the CEL/DSF/IRN combination GBM 5 had a medium response, whereas with the CEL/IRN/ITZ combination it has a high response. This is because DSF does not target any of the candidate genes associated with GBM growth that are present in GBM 5, whereas ITZ does (Table 3). When CEL is replaced with DSF (DSF/IRN/ITZ), the pattern of drug response changes again (Figure 3B). The level of response in GBM 2 decreases from medium to low and there is no response with GBM 3. This because DSF does not target any of the candidate genes present in GBM 2, while none of the drugs target the IDH1 or PIK3R1 genes, which are only targeted by CEL (Table 3). These observations make sense, as Quayle et al. demonstrated that mutations of the PIK3R1 gene promote the formation and development of gliomas and that GBM patients whose tumours carry mutant PIK3R1 alleles may benefit from treatment with inhibitors of Protein kinase B (PKB)/Akt [33]. Liu et al. have shown that CEL regulates apoptosis and autophagy via the PI3K/Akt signalling pathway in SGC-7901 gastric cancer cells [50]. Based on our data and the observations from Quayle et al. and Liu et al., CEL should be considered as a treatment option in GBM patients who have mutations in the PIK3R1 gene. 

The response for GBM 5 decreased from high to medium (Figure 3B), again as a consequence of DSF not targeting any of the candidate genes present in GBM 5 (Table 3). Once again, this data makes sense according to the literature. Only GBM 5 contained variants of the PIK3CA gene (Figure 2). Missense mutations in this gene have been shown to make GBMs more sensitive to treatment with a combination containing a phosphoinositide 3-kinase (P3K) inhibitor and a mitogen-activated protein kinase (MEK) inhibitor [34]. CEL has been shown to be both a P3K and MEK inhibitor [50,51], thus removing it from the combination reduced the combinations efficacy against GBM 5 (Figure 3B). The above observations demonstrate that by personalising the treatment through the selection of a combination of drugs based on the genetic variants present in a particular GBM patient’s tumour we can improve the level at which that GBM responds to treatment and thus potentially improve survival.

As a negative control and to demonstrate that our observations were due to personalisation and not to treating the cells with a random cocktail of drugs we tested a combination (CAP/TCP/TMZ) made up of drugs that provided no response when used individually (Figure 3B). As expected, this combination provided no response across all GBM samples except for GBM 4. However, the cell viability achieved at a concentration of log 5 nM was 48.9% and thus just below the 50% required to be considered a response. Furthermore, TMZ alone had a cell viability of 52.1% when used to treat GBM 4, therefore, the combination offered no significant benefit (*P* value = 0.12) compared to TMZ alone.

The cytotoxicity of DSF has been shown to be dependent on the presence of copper(II) (Cu) or some other transition bivalent metal ions [52,53,54,55]. Therefore, it was decided to add CoGlu to a combination of DSF and IRN to assess its influence on response rate (Figure 3B). The addition of CoGlu results in an increase in response rate across all GBM samples. GBM 1, 2, 3 and 5 saw an increase in response even though they did not respond to either CoGlu or DSF individually (Figure 3). We believe that this is due to the production of reactive oxygen species (ROS) as a result of CoGlu and DSF forming a Diethyldithiocarbomate (DDC)/Cu complex as well as the cytotoxicity associated with the DDC/Cu complex [55] rather than being associated with any specific candidate genes present in the GBM samples. The un-specific nature of CoGlu/DSF results in it inducing some level of response across all GBM samples as it does not rely on any particular gene mutation.

For our last set of combinations we included PTV. Firstly, we combined PTV with IRN, which resulted in a high response for all GBM samples except GBM 1, which had a very low response (Figure 3B). The responses are similar when compared to the drugs individually (Figure 3A). Then, we included PTV in the CEL/IRN/ITZ combination, the most promising three-drug combination. Again, there was a high response for all GBM samples except GBM 1 (Figure 3B). These observations are due mainly to PTV, which induces a high response in all GBM samples except for GBM 1 when used on its own. However, even when combined with other drugs it still does not induce a response in GBM 1 (Figure 3B). This is because only IRN targets the candidate gene PKHD1 that is present in GBM 1 (Table 3). This data further supports the personalisation of GBM treatment to a particular tumour based on the genes that are present. Finally, we decided to combine CoGlu, DSF, IRN and PTV, which as expected resulted in a high response rate for GBM samples 2, 3, 4, 5 and 6 as a result of PTV being included (Figure 3B). However, this time we achieved a medium response rate in GBM 1 as a result of the generation of ROS and the DDC/Cu complex (Figure 3B). This data would suggest that the best approach to treating GBM is personalisation combined with an un-specific treatment option such as DSF/CoGlu or PTV.

### 3.5. The Influence of Personalisation on GBM Recurrence

**Figure 4:**
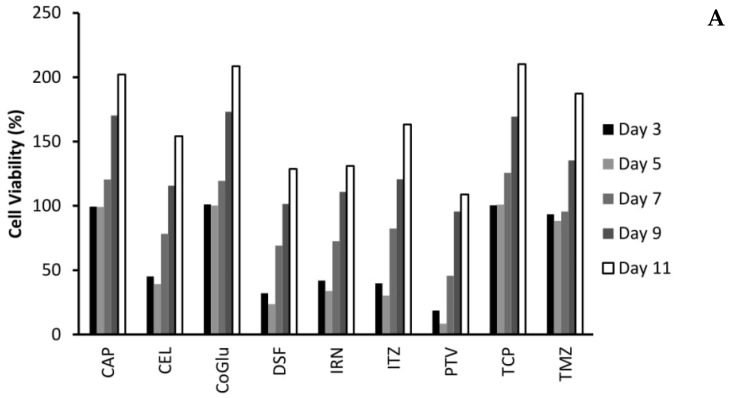

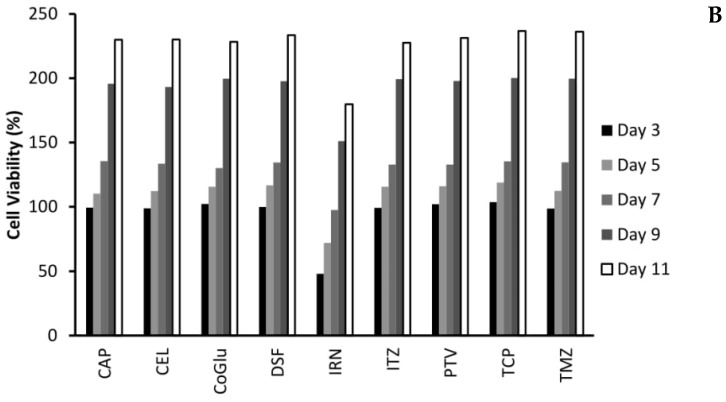
The influence of the single drugs on the recurrence rate of GBM 4 (**A**) and GBM 1 (**B**) over an 11-day period. The concentration of each drug was 5 log nM.

**Figure 5:**
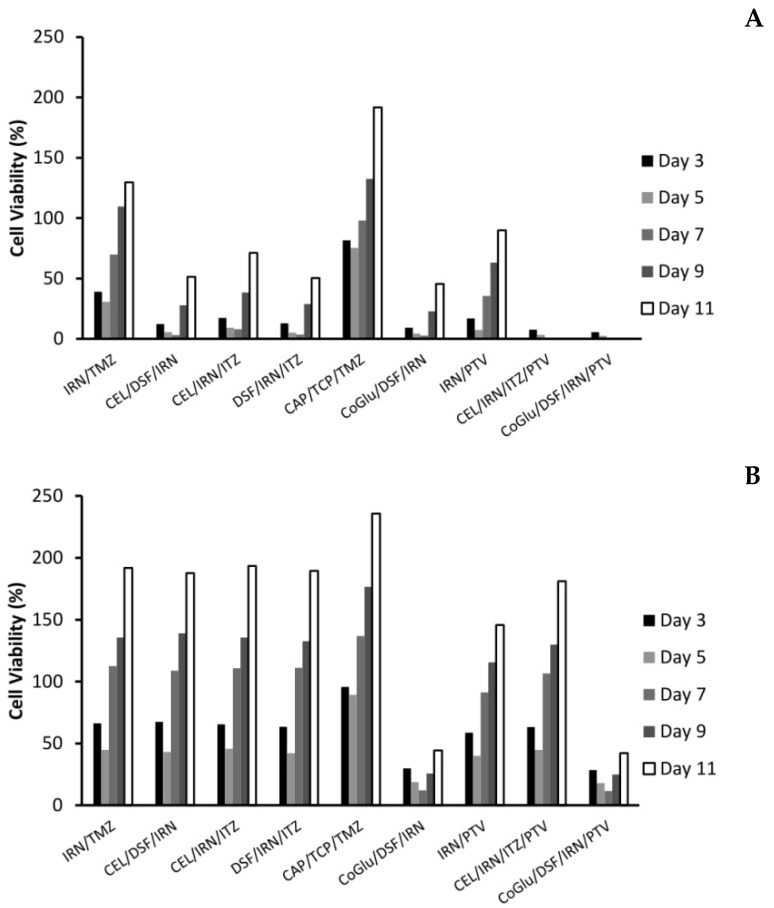
The influence of the combinations on the recurrence rate of GBM 4 (**A**) and GBM 1 (**B**) over an 11-day period. The concentration of each drug in the combination was 5 log nM.

One of the biggest issues with GBM treatment is recurrence. Therefore, to demonstrate if by personalising treatment through the selection of a combination of drugs based on the genes they target we can decrease recurrence, we evaluated the cytotoxicity of the individual drugs (Figure 4A,B) and the combinations (Figure 5A,B) over an 11-day period. We choose GBM 4 as it was the most responsive sample and GBM 1 as it was the least responsive. Figure 4A,B demonstrate that with the individual drugs we see a similar trend for all drugs across both samples. The cell viability increases significantly (*P* values < 0.04) by day 11. For example, high dose (5 log nM) CAP, CoGlu, TCP and TMZ reduced the cell viability of GBM 4 to between 88.2% and 99.1% at day 5. However, by day 11 the cell viability had increased to between 187.3% and 202.1% (Figure 4A). A similar trend was seen with high dose CEL, DSF, IRN and ITZ, with cell viability decreasing to between 23.7% and 39.2% by day 5, but increasing to between 128.7% to 163.2% by day 5 (Figure 4A). With PVT cell viability decreased to 8.5% by day 5, increasing to 108.9% by day 11 (Figure 4A). Even with drugs that have a high response rate for this particular GBM, we still see recurrence. With GBM 1 only high dose IRN was capable of providing any significant reduction (*P* value = 0.015) in cell viability, reducing it to 47.9% at day 3. However, cell viability increased to 179.7% by day 11 (Figure 4B). The other drugs had no significant (*P* values > 0.212) influence on reducing cell viability, which reached between 227.6% and 236.7% by day 11. This data demonstrates that single drug therapy will never be enough to stop or reduce recurrence in GBM treatment.

With the personalised combinations we do see a reduction in recurrence, particularly for those combinations that have a significant reduction on cell viability over the first seven days (Figure 5A,B). For example, with GBM 4 the three personalised combinations (CEL/DSF/IRN, CEL/IRN/ITZ and DSF/IRN/ITZ) reduced cell viability to 3.1%, 7.9% and 3.5% by day 7 respectively, with the cell viability increasing to 51.3%, 71.2% and 50.4% at day 11 (Figure 5A). Comparing this to the individual drugs (Figure 4A) were cell viability never went below 23% and increased to above 128%, clearly demonstrates that personalisation has the potential to be more effective, while also reducing recurrence. This is further strengthened when we compare the personalised combinations to the IRN/TMZ combination, which has been used in the clinic with promising results. The IRN/TMZ combination reduced cell viability for GBM 4 to 30.7% at day 3, which increased to 129.7% by day 11 (Figure 5A). To demonstrate that the effect on recurrence is not due to a ‘random cocktail’ of drugs, but due to personalisation, we evaluated the combination CAP/TCP/TMZ for its effect on recurrence. Figure 5A shows that this combination only reduced cell viability to 75.2%, which increased to 191.6% by day 11 clearly demonstrating that the results for the personalised combinations were not due to a random cocktail of drugs, but to personalising the treatment based on candidate genes found in the GBM.

When we look at the recurrence data for the personalised combination that includes PTV (CEL/IRN/ITZ/PTV) we see a significant (*P* Values < 0.009) improvement in efficacy and reduction in recurrence (Figure 5A). The cell viability is reduced to 0% by day 7 and remains there until day 11. We believe that this is again due to personalisation, rather than the inclusion of PVT because although the IRN/PVT combination does have improved efficacy when compared to the drugs individually (Figure 4A) the cell viability was reduced to 7.3% by day 5, but increased to 89.9% by day 11. The un-specific combination of CoGlu/DSF/IRN reduced cell viability to 2.7% by day 7, however, it did not stop recurrence as the cell viability increased to 45.5% by day 11 (Figure 5A). The CoGlu/DSF/IRN/PTV combination reduced cell viability to 0 by day 7, were it remained until day 11 (Figure 5A), demonstrating that it has the potential to be effective while also reducing recurrence.

The real test for personalisation was going to be GBM 1 which was a highly aggressive GBM that responded to none of the individual drugs, except a very low response to IRN (Figure 3A). Furthermore, it had very low responses to all of the combinations except for CoGlu/DSF/IRN and CoGlu/DSF/IRN/PTV (Figure 3B), while the recurrence data shows a similar trend (Figure 5B). Although the personalised combinations did manage to decrease cell viability to between 42.1% and 45.7% by day 5, it increased again to between 187.6% and 193.4% by day 11 (Figure 5B). The IRN/TMZ combination had a similar profile decreasing cell viability to 44.7% by day 5, which increased to 191.8% by day 11, while the random (CAP/TCP/TMZ) combination only managed to decrease cell viability to 89.2%, which then increased to 235.6% by day 11 (Figure 5B). The fact that the personalised combinations did not perform significantly better (*P* value = 0.245) than the IRN/TMZ combination in this particular sample supports the hypothesis of increased efficacy due to personalisation rather than a random cocktail of drugs. If it was due to a random cocktail of drugs, we would have expected at least one of the personalised combinations to outperform the IRN/TMZ combination.

The inclusion of PVT into the personalised combination (CEL/IRN/ITZ/PTV) did not significantly (*P* value = 0.201) improve its efficacy (Figure 5B). This is not surprising given the fact that PVT did not target any of the candidate drugs in GBM 1 (Table 3), adding further weight to the importance of personalisation. The un-specific combination of CoGlu/DSF/IRN reduced cell viability to 12.2% by day 7, however, it did not stop recurrence as the cell viability increased to 44.3% by day 11 (Figure 5B). The CoGlu/DSF/IRN/PTV combination had a similar trend reducing cell viability to 11.5% by day 7, increasing to 42.1% by day 11 (Figure 5B).

## 4. Discussion

This study clearly demonstrates that serum-cultured cells up to passage number 3 are genetically similar to the primary tumour tissue they were cultured from when considering variants likely to influence treatment response. This is significant as it supports the use of serum-cultured cell lines in these types of studies, whereas previous studies have suggested this method gives insufficient representation of tumours [56,57,58,59]. Whole Exome Sequencing identified eight promising candidate genes associated with treatment response. The genes identified were involved in transcriptional regulation (ATRX), tumour suppression (TP53, PTEN and CDKN2A), proliferation and survival (IDH1, PKHD1, PIK3R1 and PIK3CA). The cell viability data highlighted the problem with the current treatment regime for GBM and the need for improved therapy options as TMZ produced no response in any of the GBM samples, even though only GBM 2 and 4 had an unmethylated MGMT. Of the eight other drugs investigated, only five (CEL, DSF, IRN, ITZ and PTV) induced a response in any of the GBM samples. From these five drugs, it was possible to identify variant genes for three (CEL, IRN and ITZ) of them that might be involved in their response. A series of three-drug personalised combinations that targeted all or most of the eight genes above significantly improved cytotoxicity when compared to the drugs on their own or a series of control combinations. DSF and PTV were identified as working via un-specific mechanisms: the generation of ROS and the reduction in lipid levels, respectively. The inclusion of PTV into the personalised three-drug combination further increased cytotoxicity. One of the biggest issues with GBM treatment is recurrence. The use of personalised combinations and personalised combinations containing an un-specific drug such as DSF or PTV significantly reduced recurrence and in some cases stopped it all together when compared to the drugs individually and the control combinations. In this study, we did not investigate or identify each of the individual variants of a gene to see if this variant was responsible for the responsiveness of a drug. We believe that this level of detailed analysis is not needed or logistically possible when dealing with a number of different genes. What this study has demonstrated is that the choice of drug should be based on whether or not a particular gene has mutated variant, not the nature of that variant. By narrowing this to cancer driver genes, that when mutated have a high possibility of causing or enhancing tumour growth and a small group of drugs that have shown to be effective against GBM, we move closer to the possibility of personalizing treatment. We do appreciate that some of these drugs may not cross the blood brain barrier (BBB), which will be an issue for most drugs used to treat GBM. However, with improvements in convection enhanced delivery and the development of implantable devices, these combinations could be delivered directly into the tumour resection cavity thus by-passing the BBB. Furthermore, preclinical toxicity and efficacy studies would allow for an exact local dose of each drug in the combination to be determined, which will more than likely be lower than the systemic dose. This would potentially reduce the side effects associated with GBM treatment improving the patient’s quality of life. This pilot study demonstrates for the first time that whole exome sequencing has the potential to improve the treatment of GBM patients by using exonic, nonsynonymous variants in commonly mutated cancer driver genes and drug response to personalise treatment. This novel approach could potentially offer a new avenue of treatment for this terrible disease.

## Figures and Tables

**Table 1:** Pathology data for each of the six GBM tumour samples.

	Gene			
Sample	IDH	ATRX	P53	MGMT
GBM1	Wild Type	Wild Type	Wild Type	Methylated
GBM2	Wild Type	Wild Type	Unspecified	Unmethylated
GBM3	Mutated	Wild Type	Wild Type	Unspecified
GBM4	Mutated	Mutated	Mutated	Unspecified
GBM5	Wild Type	Wild Type	Wild Type	Unmethylated
GBM6	Wild Type	Wild Type	Wild Type	Unspecified

**Table 2:** Full name and function of the 13 genes associated with GBM treatment response.

Gene	Full Name	Function
ATRX	Alpha Thalassemia/Mental Retardation Syndrome X-Linked	Involved in transcriptional regulation and chromatin remodelling
USH2A	Usher Syndrome 2A	Involved in hearing and vision
TP53	Tumor Protein P53	Tumour suppressor gene.
PTEN	Phosphatase And Tensin Homolog	Tumour suppressor gene
PKHD1	Polycystic Kidney And Hepatic Disease 1	Involved in cell adhesion, repulsion and proliferation.
PIK3R1	Phosphoinositide-3-Kinase Regulatory Subunit 1	Involved in cell proliferation and survival
PIK3CA	Phosphatidylinositol-4,5-Bisphosphate 3-Kinase Catalytic Subunit Alpha	Involved in cell proliferation and survival
PCLO	Piccolo Presynaptic Cytomatrix Protein	Cell scaffolding protein
MUC17	Mucin 17	Maintains homeostasis on mucosal surfaces
MUC16	Mucin 16	Provides a protective and lubricating barrier at mucosal surfaces
IDH1	Isocitrate Dehydrogenase 1	Is necessary for many cellular processes
FLG	Filaggrin	Involved in the structure of the epidermis and plays an important role in the barrier function of the epidermis
CDKN2A	Cyclin Dependent Kinase Inhibitor 2A	Tumour suppressor gene.

**Table 3:** Summary of the genes involved with the response of each drug.

Gene	Tumour Cells	Drug
ATRX	GBM 4, 5 and 6	CEL, IRN, ITZ and PTV
TP53	GBM 4 and 5	CEL, IRN, ITZ and PTV
PTEN	GBM 5	CEL, IRN, ITZ and PTV
PKHD1	GBM 1, 4 and 6	IRN
PIK3R1	GBM 3	CEL and PTV
PIK3CA	GBM 5	CEL, IRN, ITZ and PTV
IDH1	GBM 3 and 4	CEL and PTV
CDKN2A	GBM 5	CEL, IRN, ITZ and PTV
